# Clinical Effectiveness of Nasal Dilators in Sleep-Disordered Breathing: A Systematic Review and Meta-Analysis

**DOI:** 10.7759/cureus.100663

**Published:** 2026-01-03

**Authors:** Sultan Alotaibi, Bushra Wadi Bin Saddiq, Retaj Sanad Alfarhoud, Saud Turki N. Alghamdi, Amina Jaafar Alsaffar, Abdulaziz Mohammad Alnabhan, Moussa A Alkhateeb, Kawthar Hassan Albahrani, Shahad Ahmed Alowibidy, Zahra Ibrahim Almahdi, Hanin Abdulrahim Almaghrabi, Majed Alnabulsi

**Affiliations:** 1 General Medicine, General Medicine Practice Program, King Saud Bin Abdulaziz University for Health Sciences, Riyadh, SAU; 2 Medical College, Batterjee Medical College, Jeddah, SAU; 3 General Medicine, General Medicine Practice Program, King Abdulaziz University, Jeddah, SAU; 4 Faculty of Medicine, Al-Baha University, Al-Baha, SAU; 5 General Medicine, General Medicine Practice Program, Mansoura University, Mansoura, EGY; 6 General Medicine, General Medicine Practice Program, King Saud Bin Abdulaziz University for Health Sciences, Jeddah, SAU; 7 Internal Medicine, Faculty of Medicine, King Abdulaziz University, Jeddah, SAU; 8 General Medicine, General Medicine Practice Program, Imam Abdulrahman Bin Faisal University, Dammam, SAU; 9 Respiratory Therapy, College of Applied Medical Sciences, King Saud Bin Abdulaziz University for Health Sciences, Jeddah, SAU; 10 Respiratory Therapy, King Saud Bin Abdulaziz University for Health Sciences, Jeddah, SAU; 11 Internal Medicine, Medicine Program, Batterjee Medical College, Jeddah, SAU

**Keywords:** continuous positive airway pressure, dilators, nasal obstruction treatment, obstructive sleep apnea and snoring syndrome, stents

## Abstract

Background: Nasal dilators are widely used as a noninvasive intervention for snoring and obstructive sleep apnea (OSA), yet their objective effects on sleep-disordered breathing and polysomnographic outcomes remain uncertain. This systematic review and meta-analysis aim to assess the effectiveness of nasal dilators in managing sleep-disordered breathing.

Methods: Scopus, PubMed, Web of Science, and ProQuest were systematically searched from inception to January 2024 for studies assessing the efficacy of internal or external nasal dilators in adult patients with sleep-disordered breathing. The standardized mean difference (SMD) was used to pool and compare the findings across studies using a random-effects model.* *

Results:* *Our search strategy yielded 290 records, of which 17 studies with a pooled population size of 496 participants were found to be eligible for inclusion. We did not find any significant differences in apnea-hypopnea index, apnea index, hypopnea index, snoring index, sleep time, sleep architecture, REM latency, oxygen saturation, and nasal airway resistance between participants who received nasal dilators and control subjects.

Conclusions: Nasal dilators cannot be recommended as monotherapy for sleep-disordered breathing. However, it may be helpful as adjunctive therapy in specific populations with mild symptoms or nasal congestion.

## Introduction and background

Obstructive sleep apnea (OSA) is a disorder characterized by periodic interruptions of breathing during sleep, resulting from the total or partial collapse of the upper airway, which leads to apnea or hypopnea [1,2]. Epidemiological studies indicate that OSA affects approximately 9% to 38% of adults worldwide, based on diagnostic criteria using an apnea-hypopnea index (AHI) threshold of ≥5 events per hour [3]. The prevalence is typically higher among men, those with a body mass index above normal, and geriatric populations [3]. The symptomatology of OSA is largely non-pathognomonic and typically includes snoring, frequent apneas, sleep fragmentation, excessive daytime sleepiness, disruptions in gas exchange, and fatigue [4]. These symptoms worsen the consequences of sleep apnea on the cardiovascular system, metabolism, inflammation, and neurocognition [2,4].

Continuous positive airway pressure (CPAP) is considered the gold standard treatment for managing patients with OSA [5]. Despite the benefits of CPAP, including reduced daytime sleepiness and improvements in memory, executive function, overall daytime function, and cardiovascular outcomes [5], adherence to CPAP therapy remains a challenge, with 30%-40% of patients being non-adherent [6]. The reasons for non-adherence to CPAP included physical discomfort with the mask, dryness or congestion, nasal resistance, claustrophobia, high cost, and device maintenance [5,7]. Thus, several alternative modalities have been developed for managing non-adherent patients, including mandibular advancement devices, surgery, hypoglossal nerve stimulation, nasal expiratory positive airway pressure (EPAP) devices, and oral pressure therapy [5].

Nasal dilators are non-invasive, easy-to-use, and cost-effective devices originally developed to reduce nasal airflow resistance and improve nasal patency, rather than to treat OSA. These consist of products that can be placed internally (internal nasal dilators) or externally (external nasal dilators), to mechanically enlarge the nasal passages and improve nasal patency [8]. External nasal dilators expand the cross-sectional area of the nasal valve, thereby reducing nasal resistance and stabilizing the lateral nasal vestibule to prevent collapse during inspiration [9]. In contrast, internal nasal dilators are placed within the nostrils to mechanically support the internal nasal valve region and prevent nasal wall collapse, thereby enhancing nasal breathing and reducing airflow resistance [10]. Importantly, nasal airflow resistance represents an upstream, relatively fixed component of the upper airway, whereas OSA is primarily driven by dynamic collapse of the pharyngeal airway during sleep. Consequently, although improving nasal patency may modify airflow distribution or breathing route, it does not directly address the pharyngeal collapsibility that underlies OSA, and nasal dilators should therefore not be considered mechanistically equivalent to established OSA therapies [1,2,8].

Several studies have investigated the efficacy of internal and external nasal dilators, suggesting that these devices may relieve some symptoms of OSA. For instance, Gelardi et al. [11] demonstrated that Nas-air® (E.P. Medica Srl, Fusignano, Italy) internal nasal dilators and Breathe Right® (Foundation Consumer Healthcare, LLC, Pittsburgh, PA) external nasal dilators reduced snoring time among patients who snore compared to baseline, with a significant difference between the two modalities. However, only Nas-air® internal nasal dilators significantly improved sleep visual analog scale score, indicating an improvement in sleep quality [11]. In addition, the group demonstrated that Nas-air® internal nasal dilators significantly reduced AHI, oxygen desaturation index, and sleep scores compared to baseline among patients diagnosed with OSA [10,12]. In contrast, earlier studies by Hoffstein et al. [13] showed that the internal nasal dilator (NoZovent™) did not affect the number of apneas, hypopneas, oxygen saturation, or snoring parameters during stages I, II, and IV of sleep, although these parameters were significantly reduced during stage II slow-wave sleep.

Only one meta-analytic study has assessed the effects of internal and external nasal dilators on patients with OSA, with no clinically significant changes in AHI, apnea index, respiratory disturbance index, snoring index, sleep architecture, lowest oxygen saturation, or daytime sleepiness [14]. However, all studies included in the meta-analysis by Camacho et al. [14] were published between 1992 and 2012, and did not include more recent studies such as those by Ieto et al. [15], Wheatley et al. [16], or Maxwell et al. [17]. Thus, this study aims to conduct an up-to-date systematic review and meta-analysis to assess the effectiveness of internal and external nasal dilators in managing snoring and OSA, incorporating studies published in the past decade.

## Review

Methodology

This study was conducted in accordance with the Preferred Reporting Items for Systematic Reviews and Meta-Analyses (PRISMA) guidelines [18]. The study protocol was registered in PROSPERO [19] with the following ID: CRD42024627324.

Data Sources and Search Strategy

Scopus, PubMed, Web of Science, and ProQuest databases were systematically searched from database inception till January 2024 using the following Medical Subject Headings (MeSH) terms: ("Obstructive sleep apnea" OR "OSA" OR "sleep disordered breathing" OR "sleep apnea syndromes" OR "upper airway resistance syndrome") AND ("nasal dilator" OR "nasal strips" OR "Breathe Right Strips" OR "NoZovent" OR "nasal expanders" OR "nasal breathing aids" OR "nasal stents" OR "dilator strips" OR "Breathe strips" OR "nasal breathing strips" OR "external nasal dilators" OR "nasal band strips" OR "nasal valve dilators" OR "nasal congestion strips" OR "nasal expander strips" OR "nasal airflow strips" OR "nasal passage openers" OR "nose opening strips" OR "nasal valve support strips")

Additionally, an extensive manual search was conducted throughout the study period to identify any studies that may have been overlooked. No search limits or filters were applied.

Selection Criteria

We included randomized controlled trials (RCTs) and observational studies assessing the efficacy of either external nasal dilators, such as Breathe Right Strips, or internal nasal dilators, such as NoZovent (NoZovent AB, Habo, Sweden), in adult patients diagnosed with sleep-disordered breathing, including primary snorers and patients with OSA. In addition, the use of baseline (pretreatment) polysomnographic or sleep study data to assess results before and after the use of nasal dilators was required for studies to be eligible for inclusion. Studies focusing on the pediatric population, or patients with central sleep apnea, or combined treatments were excluded. We also eliminated case reports, abstracts, reviews, commentaries, and studies that were not in English.

Data Extraction

All records retrieved from the database search were exported into EndNote software, and duplicates were removed. Two authors (KA and ZA) independently and blindly evaluated the studies based on their titles and abstracts against our inclusion criteria. Subsequently, the same two authors conducted a more comprehensive full-text screening of the listed studies. Any disagreements were resolved through the involvement of a third reviewer.

Study Outcomes

We compared the effects of nasal strips with those of the control group on the following 11 study outcomes: AHI, apnea index, hypopnea index, snoring index, total sleep time (TST), percentage of time spent in the four sleep stages, rapid eye movement (REM) latency, Epworth Sleepiness Scale (ESS) score [20], minimum and mean oxygen saturation, and nasal airway resistance.

Quality Assessment

Two authors (SA and RA) independently assessed the quality of the included studies, and any disagreements were resolved by consulting with the third author. The NIH tool was used to assess the quality of single-arm studies, while the RoB2 tool was used for randomized trials [21].

Data Synthesis and Statistical Analysis

Continuous outcomes were extracted and summarized as means and standard deviations (SDs). When studies reported medians with interquartile ranges (IQRs) or ranges, established Cochrane recommendations were applied to estimate means and SDs [22]. Quantitative synthesis was conducted using random-effects models to account for between-study variability.

Analyses were performed separately according to study design. RCTs, including parallel-group and crossover designs, were synthesized independently from uncontrolled pre-post and single-arm studies. For RCTs, mean differences (MDs) with corresponding 95% confidence intervals (CIs) were calculated to compare nasal dilators with control conditions. Crossover trials were handled cautiously in accordance with Cochrane guidance, using within-participant comparisons when appropriate and avoiding double-counting of participants.

For uncontrolled pre-post and single-arm studies, a separate single-arm meta-analysis was conducted, pooling pre-post mean differences within the intervention arm only. These analyses were not combined with controlled trials and were interpreted descriptively, recognizing their inherently lower level of evidence.

Between-study heterogeneity was assessed using the I² statistic, with values above 50% indicating substantial heterogeneity and values above 75% indicating considerable heterogeneity [23]. All analyses were performed using the meta package in R software (version 4.2.1) [24,25].

Results

**Figure 1:**
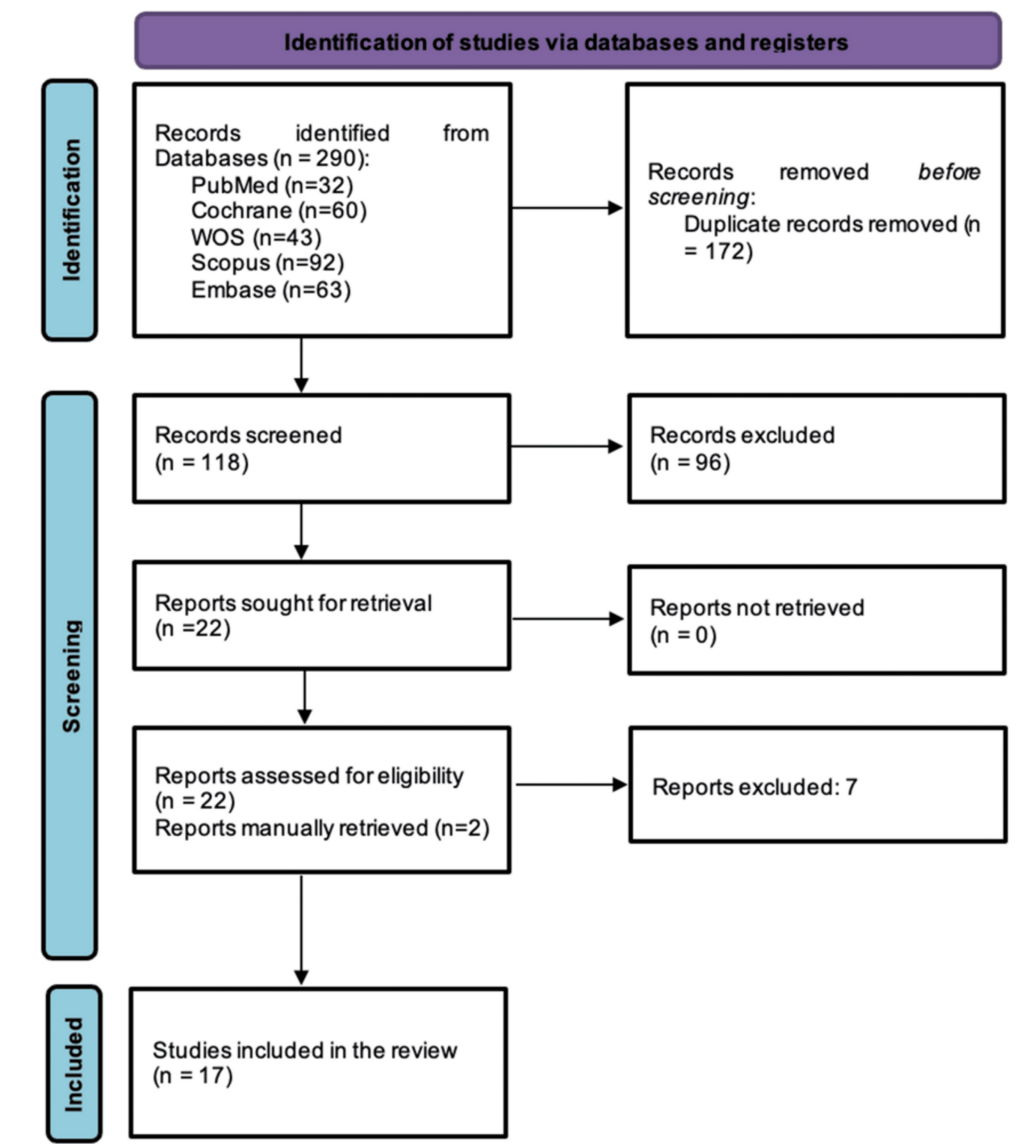
PRISMA flow diagram of the study selection process. PRISMA, Preferred Reporting Items for Systematic Reviews and Meta-Analyses

Our search strategy yielded 290 records, of which 172 were duplicates. The remaining 118 records were screened based on title and abstract, and 96 were excluded because they did not meet our inclusion criteria. The full text of 22 articles was retrieved and screened, of which 15 met the eligibility criteria for inclusion. Two additional eligible articles were identified through a manual search, bringing the total number of included studies to 17 [13,15-17,26-38]. The PRISMA flowchart illustrating the study screening and selection process is presented in Figure 1.

Study Characteristics

The included studies were conducted in eight countries, with most coming from Canada (*n* = 5), followed by the United States (*n* = 3), Brazil (*n *= 2), Belgium (*n *= 2), and one each from Australia, Germany, Norway, and Sweden; one study was a multi-centered trial conducted in Germany and the United States [28]. Seven of the 17 studies used a crossover design (41.2%) [13,16,26-28,30,37], five used a pre-post design (29.4%) [29,31,32,34,38], and five were RCTs (29.4%) [15,17,33,35,36]. The population size varied between 9 and 97 participants across the included studies (median (IQR) = 16.5 (10-39)). Collectively, the studies provided data from 496 participants for our meta-analysis. A detailed summary of the studies included in the current systematic review and meta-analysis is presented in Table 1, and the participant characteristics are presented in Table 2.

**Table 1:** Summary of the included studies. AHI, apnea-hypopnea index; CAP, cyclic alternating pattern; CPAP, continuous positive airway pressure; OSA, obstructive sleep apnea; RDI, respiratory disturbance index; REM, rapid eye movement; SDB, sleep-disordered breathing; SI, snoring index; UARS, upper airway resistance syndrome

Study ID	Country	Study design	Sample size	Patients	Main findings
Amaro et al. [25]	Brazil	Randomized crossover study	12	Patients with acromegaly and moderate to severe conditions.	Nasal dilator strips show a negligible effect on obstructive sleep apnea severity in patients with acromegaly and moderate to severe conditions.
Bahammam et al. [26]	Canada	Double blind, randomized crossover	18	Patients with Upper Airway Resistance Syndrome (UARS)	Nasal dilation reduced stage 1 sleep and desaturation time, although neither the arousal index nor the AHI was significantly impacted. SDB was significantly affected by sleep stage and position.
Djupesland et al. [27]	Norway	Randomized crossover	18	Heavy snorers	Improved oxygen saturation was observed in the subgroup with severe obstruction, with no change in snoring.
Gosepath et al. [28]	Germany/USA	Pre-post interventional study	26	OSA/snoring patients with nasal obstruction	Nasal strips decreased RDI in 19 out of 26 patients, particularly those with nasal valve abnormalities. The snoring index was unaffected.
Hoffstein et al. [13]	Canada	Randomized crossover	15	Non-apneic snorers	Nasal dilation reduced snoring only in slow-wave sleep; no effect on apneas or oxygen saturation
Hoijer et al. [29]	Sweden	Randomized crossover	10	Habitual snorers/OSA patients	The nasal dilator reduced the apnea index by 47% and improved oxygen saturation. The snoring noise decreased.
Ieto et al. [15]	Brazil	Randomized controlled trial	39	Patients with primary snoring or mild-to-moderate OSA	Oropharyngeal exercises reduced the frequency and intensity of snoring; however, nasal dilator strip users did not exhibit a significant reduction in snoring.
Kerr et al. [30]	Canada	Pre-post interventional	10	Adults with OSA, some with chronic nasal obstruction, and elevated nasal resistance	Vasoconstrictors and nasal stents reduced nasal resistance by approximately 73% and decreased sleep arousals; however, they did not affect the severity of apnea, oxygen levels, or the overall quality of sleep.
Liistro et al. [31]	Belgium	Pre-post interventional study	10	Non-apneic snorers	Breathe Right strips did not improve sleep quality or reduce snoring, even in subjects with nasal valve anomalies.
Maxwell et al. [17]	USA	Randomized controlled trial	54	Pregnant women with snoring	In the absence of objective improvement in sleep-disordered breathing, the nasal dilation strip is suitable as a placebo.
McLean et al. [32]	Canada	Randomized single-blind crossover	10	OSA patients with nasal obstruction	Reduced mouth breathing; modest AHI reduction.
Metes et al. [33]	Canada	Pre-post interventional study	10	Heavy snorers	Nasal dilation improved nasal resistance but did not affect snoring, apneas, or oxygen desaturation.
Pevernagie et al. [34]	Belgium	Randomized controlled trial	12	Chronic rhinitis patients	Reduced snoring frequency; no effect on AHI.
Redline et al. [35]	USA	Randomized controlled trial	97	Patients with Mild sleep-disordered breathing (RDI 5-30)	CPAP improved well-being more than nasal dilation therapy (49% vs. 26%). No significant change in sleepiness
Scharf et al. [36]	USA	Single-blind crossover	9	Non-apneic snorers	Nasal dilation reduced cyclic alternating pattern (CAP) rates, suggesting improved sleep stability.
Schonhofer et al. [37]	Germany	Pre-post interventional	21	Moderate to severe OSA patients	No effect on RDI or SI; mild subjective snoring reduction.
Wheatley et al. [16]	Australia	Randomized crossover	70 in active phase, 55 in crossover phase	Patients with chronic nocturnal nasal congestion and sleep difficulties	Subjective improvements in nasal breathing and sleep quality; 39.1% reduction in nasal resistance during sleep; no significant change in objective snoring or AHI

**Table 2:** Baseline characteristics of participants in the included studies.

Study ID	Age (SD) (years)	Sex male (%)	BMI	Neck circumference (cm)
Amaro et al. [25]	52 (8)	8 (66.67)	33.5 (4.6)	42 (3)
Bahammam et al. [26]	46.3 (8.7)	12 (66.67)	29 (7.4)	NA
Djupesland et al. [27]	51 (7.8)	13 (72.2)	26.1 (3.5)	NA
Gosepath et al. [28]	52 (11)	NA	28.6 (5)	NA
Hoffstein et al. [13]	49 (10)	9 (60)	36 (12)	NA
Hoijer et al. [29]	47 (8.3)	6 (60)	Mean weight = 80.6 kg (range: 66.5-103.5 kg)	NA
Ieto et al. [15]	45 (13)	11 (55)	28.3 (2.5)	38 (3.5)
Kerr et al. [30]	NA	NA	NA	NA
Liistro et al. [31]	48 (12.1)	9 (90)	30 (6.4)	NA
Maxwell et al. [17]	32.6 (5.3)	0 (0)	37.9 (7.4)	NA
McLean et al. [32]	46 (5)	9 (90)	27 (1.5)	NA
Metes et al. [33]	Nasal resistance group: 13:87 years, polysomnography group: NA	Nasal resistance group: 46 (63.8)	NA	NA
Pevernagie et al. [34]	43 (2.8)	11 (91.67)	25.1 (0.8)	NA
Redline et al. [35]	49.2 (10.5)	20 (44)	32. (8.5)	NA
Scharf et al. [36]	NA	NA	NA	NA
Schonhofer et al. [37]	54.8 (11.3)	22 (84.6)	31.6 (5.7)	NA
Wheatley et al. [16]	48.5 (14.7)	47 (66.2)	28.7 (5.2)	39.1 (3.9)

Risk of Bias

**Figure 2:**
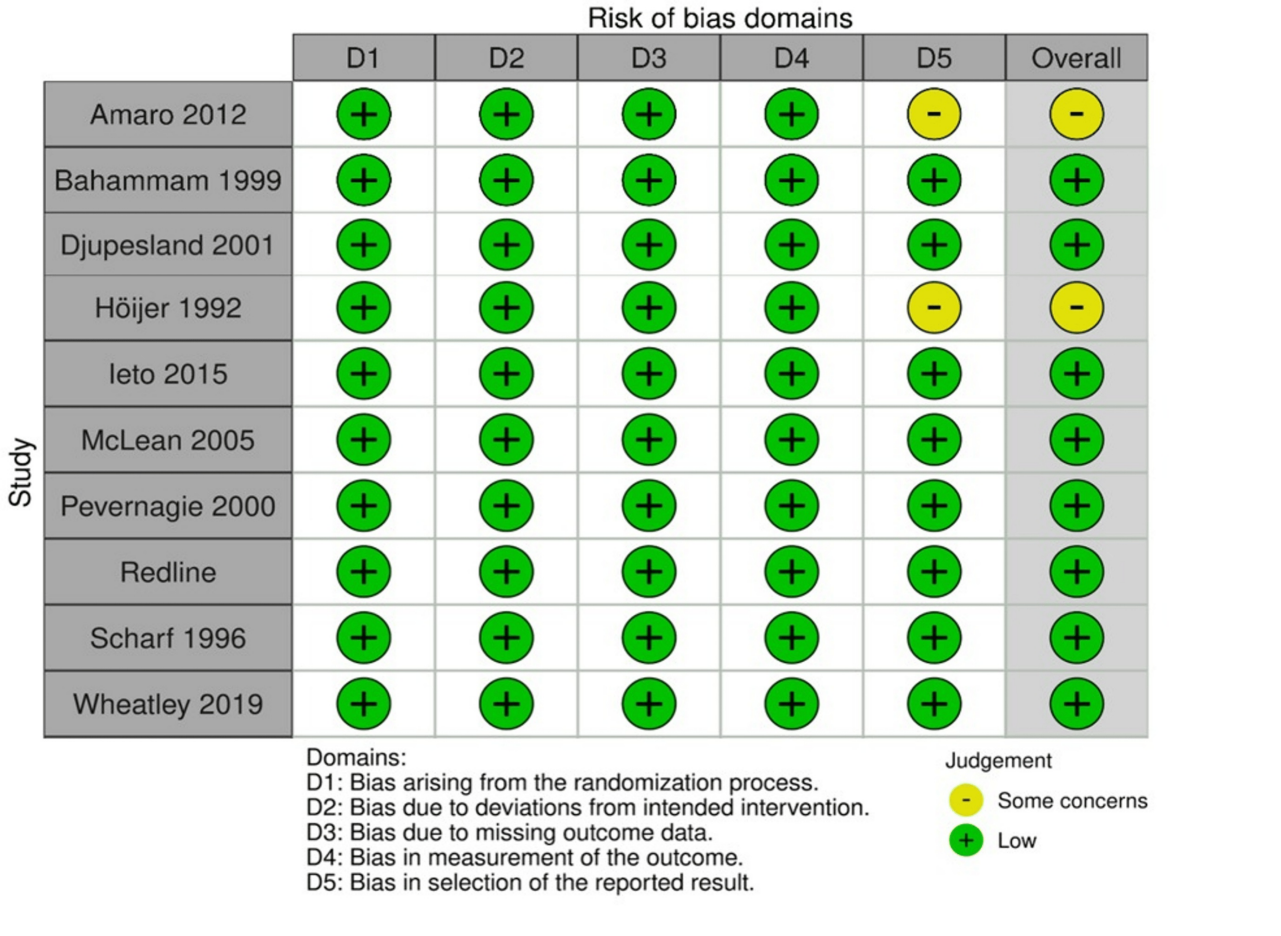
Risk of bias in randomized controlled trials assessed using the ROB2 tool. ROB2, revised Cochrane risk-of-bias tool

According to the Cochrane RoB2 tool, eight studies were categorized as having low risk of bias, while two exhibited some concerns, as outlined in Figure 2. The NIH tool for single-arm studies classified four studies as having good quality and three as having fair quality, as presented in Table 3.

**Table 3:** Risk-of-bias assessment for single-arm studies using the National Institutes of Health (NIH) tool.

Study ID	Was the research question or objective in this paper clearly stated?	Were the eligibility/selection criteria for the study population prespecified and clearly described?	Were the participants in the study representative of those who would be eligible for the test/service/intervention in the general or clinical population of interest?	Were all eligible participants who met the prespecified entry criteria enrolled?	Was the sample size sufficiently large to provide confidence in the findings?	Was the test/service/intervention clearly described and delivered consistently across the study population?	Were the outcome measures prespecified, clearly defined, valid, reliable, and assessed consistently across all study participants?	Were the people assessing the outcomes blinded to the participants' exposures/interventions?	Was the loss to follow-up after baseline 20% or less? Were those lost to follow-up accounted for in the analysis	Were outcome measures of interest taken multiple times before the intervention and multiple times after the intervention (i.e., did they use an interrupted time-series design)?	If the intervention was conducted at a group level (e.g., a whole hospital, a community, etc.), did the statistical analysis take into account the use of individual-level data to determine effects at the group level?	Did the statistical methods examine changes in outcome measures from before to after the intervention? Were statistical tests done that provided p-values for the pre-to-post changes?	Final Assessment Quality
Gosepath et al. (1999) [28]	Yes	Yes	Yes	Yes	No	Yes	Yes	No	Yes	No	Yes	Yes	Good
Hoffstein et al. (1993) [13]	Yes	Yes	Yes	Yes	No	Yes	Yes	No	Yes	No	Yes	Yes	Good
Liistro et al. (1998) [31]	Yes	No	Yes	Yes	No	Yes	Yes	No	Yes	No	Yes	No	Fair
Metes et al. (1992) [33]	Yes	Yes	Yes	Yes	No	Yes	Yes	No	Yes	No	Yes	No	Fair
Maxwell et al. (2022) [17]	Yes	Yes	Yes	Yes	Yes	Yes	Yes	No	Yes	No	Yes	Yes	Good
Schönhofer et al. (2000) [37]	Yes	Yes	Yes	Yes	No	Yes	Yes	No	No	No	Yes	No	Fair
Kerr et al. (1992) [30]	Yes	Yes	Yes	Yes	No	Yes	Yes	No	Yes	No	Yes	Yes	Good

Double-arm meta-analysis

Sleep-Disordered Breathing Severity Outcomes

**Figure 3:**
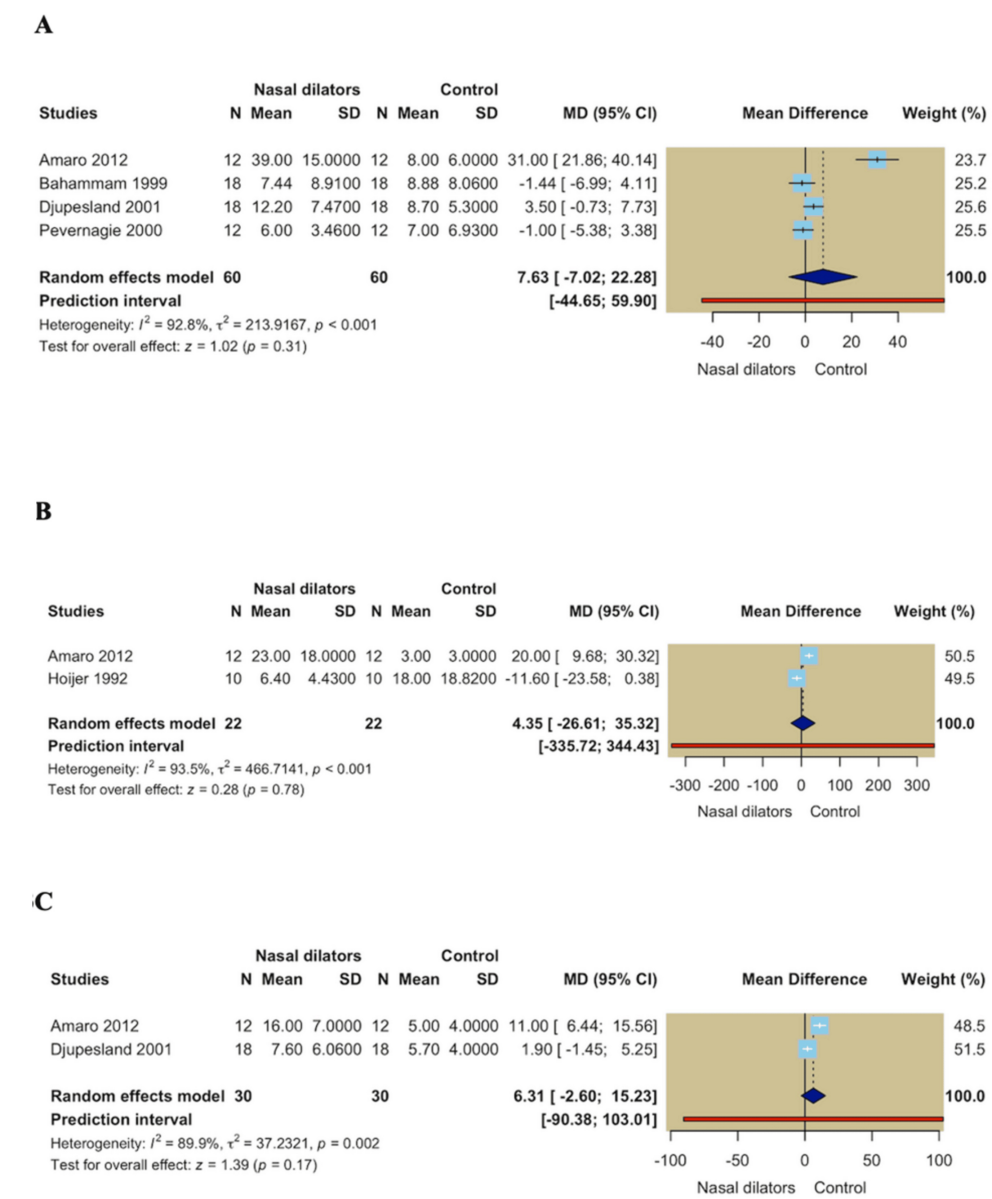
(A) Forest plot of the apnea-hypopnea index (AHI); (B) forest plot of the apnea index; (C) forest plot of the hypopnea index.

AHI: Four studies evaluated the effect of nasal dilators on the apnea-hypopnea index, encompassing 120 participants in both the nasal dilator and control groups. The pooled analysis indicated no statistically significant difference between the two groups in terms of AHI (MD = 7.63; 95% CI -7.02 to 22.28; *P* = 0.31). Significant heterogeneity was observed across the pooled studies (*I*² = 92.8%, *P* < 0.001) (Figure 3A).

Apnea Index

Two studies comprising 22 participants per group assessed the apnea index. The pooled estimate showed no significant difference between nasal dilators and control interventions in apnea index (MD = 4.35; 95% CI -26.61 to 35.32; *P* = 0.78), with substantial heterogeneity across studies (*I*² = 93.5%, *P* < 0.001) (Figure 3B).

Hypopnea Index

Two studies involving 30 participants per group evaluated the hypopnea index. The random-effects meta-analysis revealed no statistically significant improvement associated with nasal dilator use compared with control interventions (MD = 6.31; 95% CI -2.60 to 15.23; *P* = 0.17). Notably, there was a significant heterogeneity across the pooled studies (*I*² = 89.9%, *P* = 0.002) (Figure 3C).

Sleep Architecture and Continuity Outcomes

**Figure 4:**
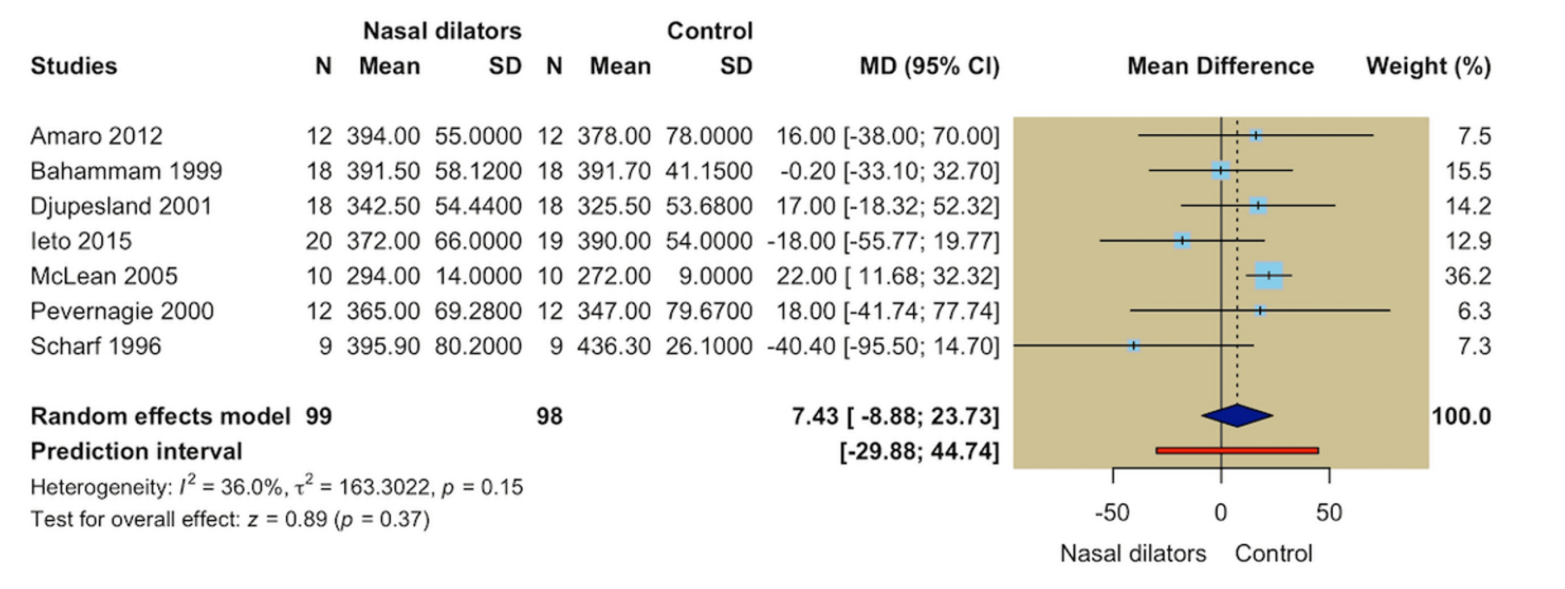
Forest plot of total sleep time.

TST: Seven studies evaluated TST, including 99 participants in the nasal dilator group and 98 participants in the control group. The random-effects meta-analysis revealed no statistically significant difference between nasal dilators and control interventions in TST (MD = 7.43; 95% CI -8.88 to 23.73; *P* = 0.37). There was a moderate heterogeneity across studies (*I*² = 36.0%, *P* = 0.15) (Figure 4).

Sleep Stages

**Figure 5:**
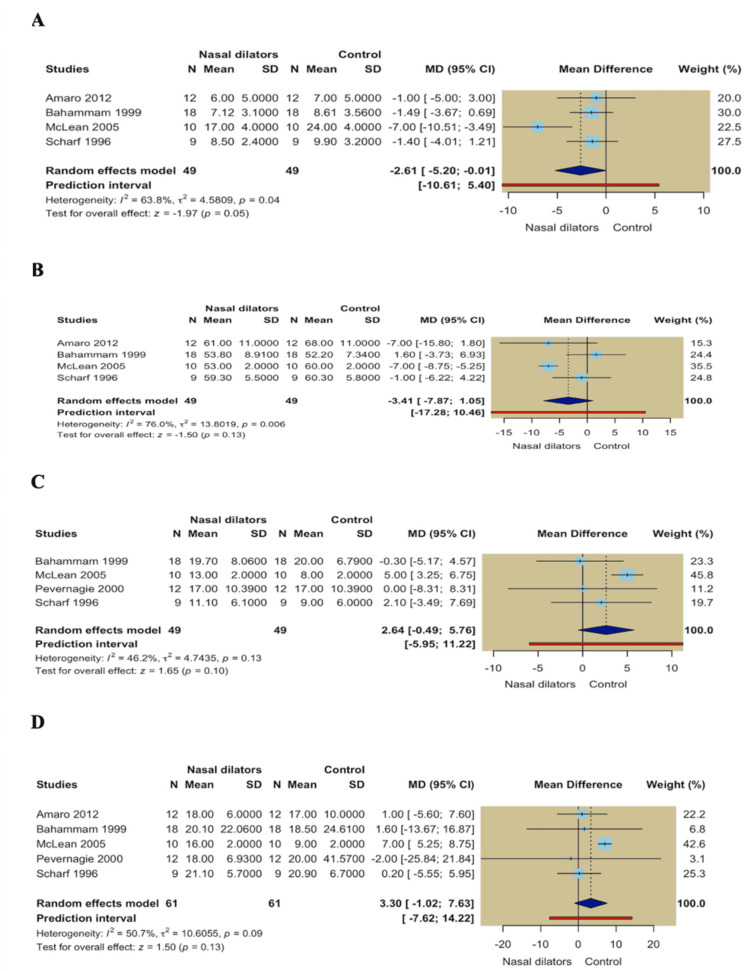
(A) Forest plot of Stage 1 sleep; (B) Forest plot of Stage 2 sleep; (C) Forest plot of Stage 3-4 sleep; (D) Forest plot of REM sleep percentage. REM, rapid eye movement

Nasal dilators showed no meaningful changes in sleep stage distribution across the pooled studies. No statistically significant differences were observed for stage 1 (MD = -2.61; 95% CI -5.20 to -0.01; *P* = 0.05), stage 2 (MD = -3.41; 95% CI -7.87 to 1.05; *P* = 0.13), slow-wave sleep stages 3-4 (MD = 2.64; 95% CI -0.49 to 5.76; *P* = 0.10), or REM sleep percentage (MD = 3.30; 95% CI -1.02 to 7.63; *P* = 0.13) (Figures 5A-5D).

REM Sleep Latency

**Figure 6:**
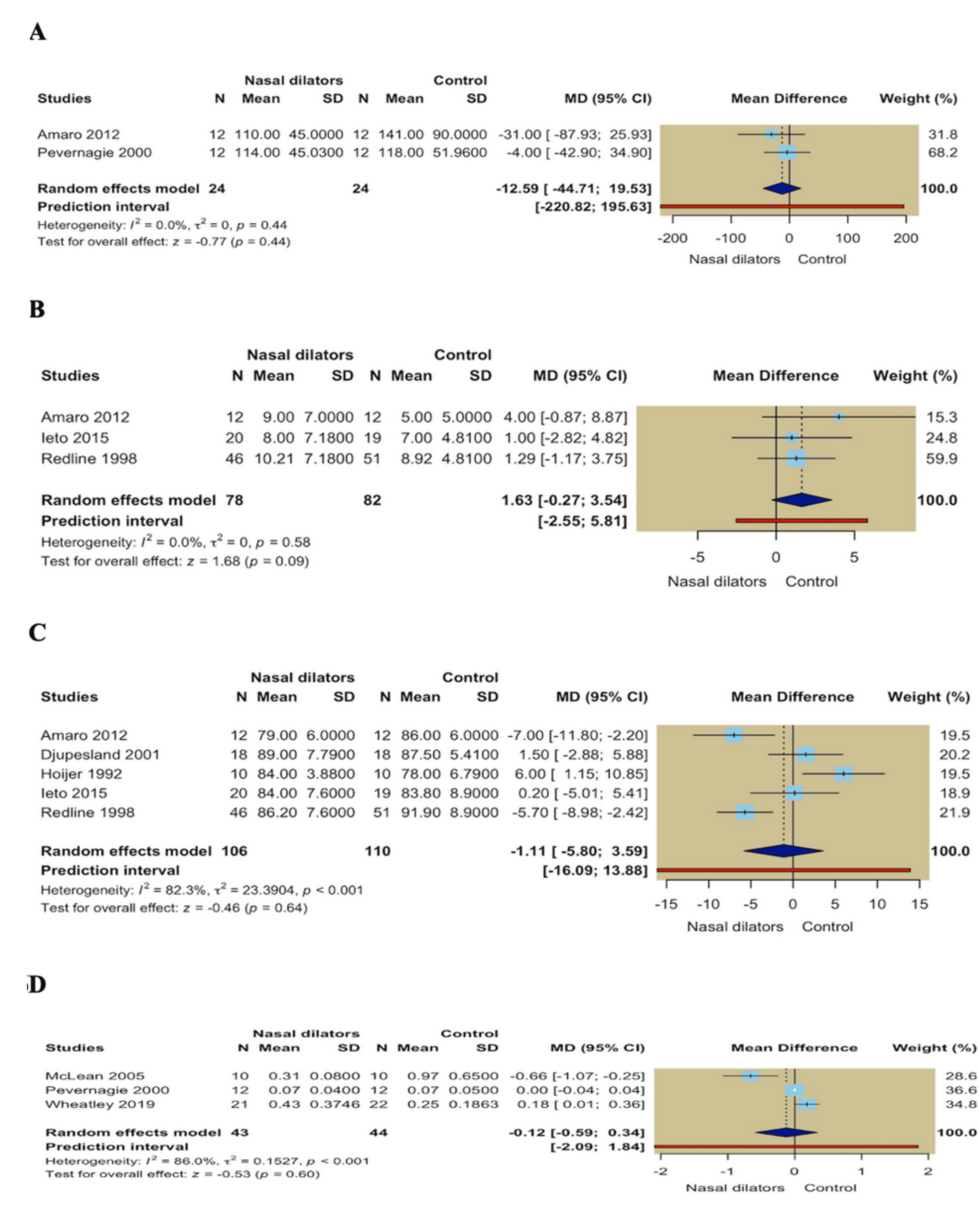
(A) Forest plot of REM latency; (B) Forest plot of Epworth Sleepiness Scale (ESS); (C) Forest plot of minimum oxygen saturation; (D) Forest plot of nasal airflow resistance. REM, rapid eye movement

Two studies comprising 24 participants per group reported REM sleep latency. The random-effects meta-analysis showed no significant difference between nasal dilators and control interventions (MD = -12.59; 95% CI -44.71 to 19.53; *P* = 0.44), with no observed considerable heterogeneity (*I*² = 0.0%; *P* = 0.44) (Figure 6A).

Other Outcomes

Daytime sleepiness (ESS): Three studies reported daytime sleepiness using the ESS, with 78 participants in the nasal dilator group and 82 in the control group. No statistically significant difference was observed between nasal dilators and control interventions (MD = 1.63; 95% CI -0.27 to 3.54; *P* = 0.09). Moreover, no heterogeneity was observed across studies (*I*² = 0.0%; *P* = 0.58) (Figure 6B).

Minimum oxygen saturation: Five studies reported the minimum nocturnal oxygen saturation, with 106 participants in the nasal dilator group and 110 in the control group. The pooled analysis showed no significant difference between the two groups in minimum oxygen saturation (MD = -1.11; 95% CI -5.80 to 3.59; *P* = 0.64). Considerable heterogeneity was present among studies (*I*² = 82.3%; *P* < 0.001) (Figure 6C).

Nasal airflow resistance: Three studies evaluated nasal airflow resistance, with 43 participants in the nasal dilator group and 44 in the control group. The pooled random-effects analysis revealed no statistically significant difference between nasal dilators and control interventions (MD = -0.12; 95% CI -0.59 to 0.34; *P* = 0.60), with substantial heterogeneity observed across studies (*I*² = 86.0%; *P* < 0.001) (Figure 6D).

**Figure 7:**
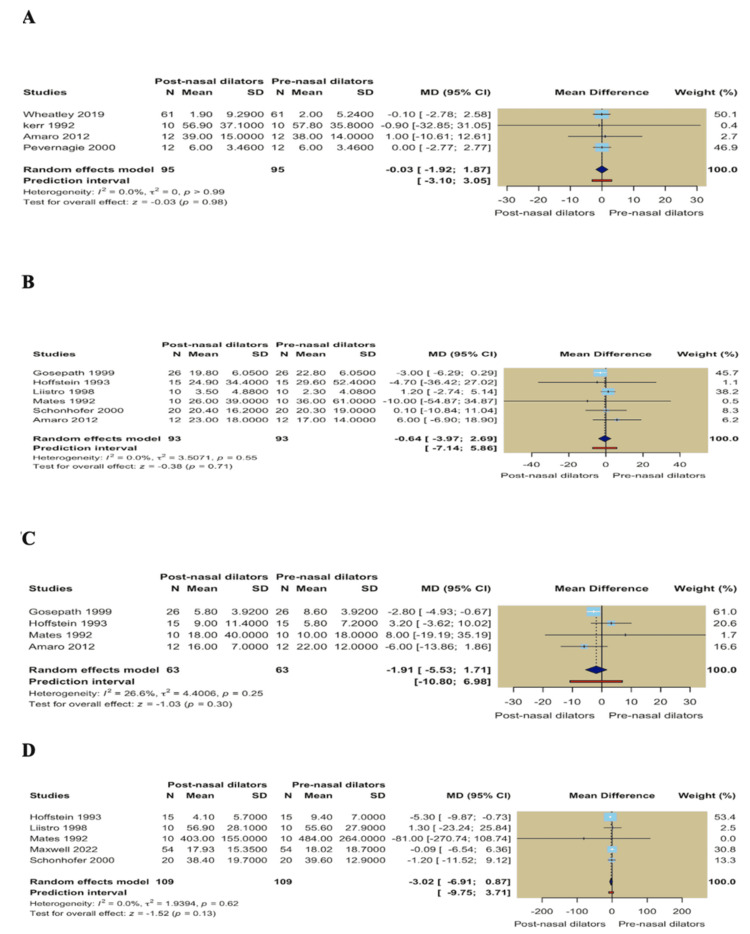
(A) Forest plot of apnea-hypopnea index; (B) Forest plot of apnea index; (C) Forest plot of hypopnea index; (D) Forest plot of snoring index.

**Figure 8:**
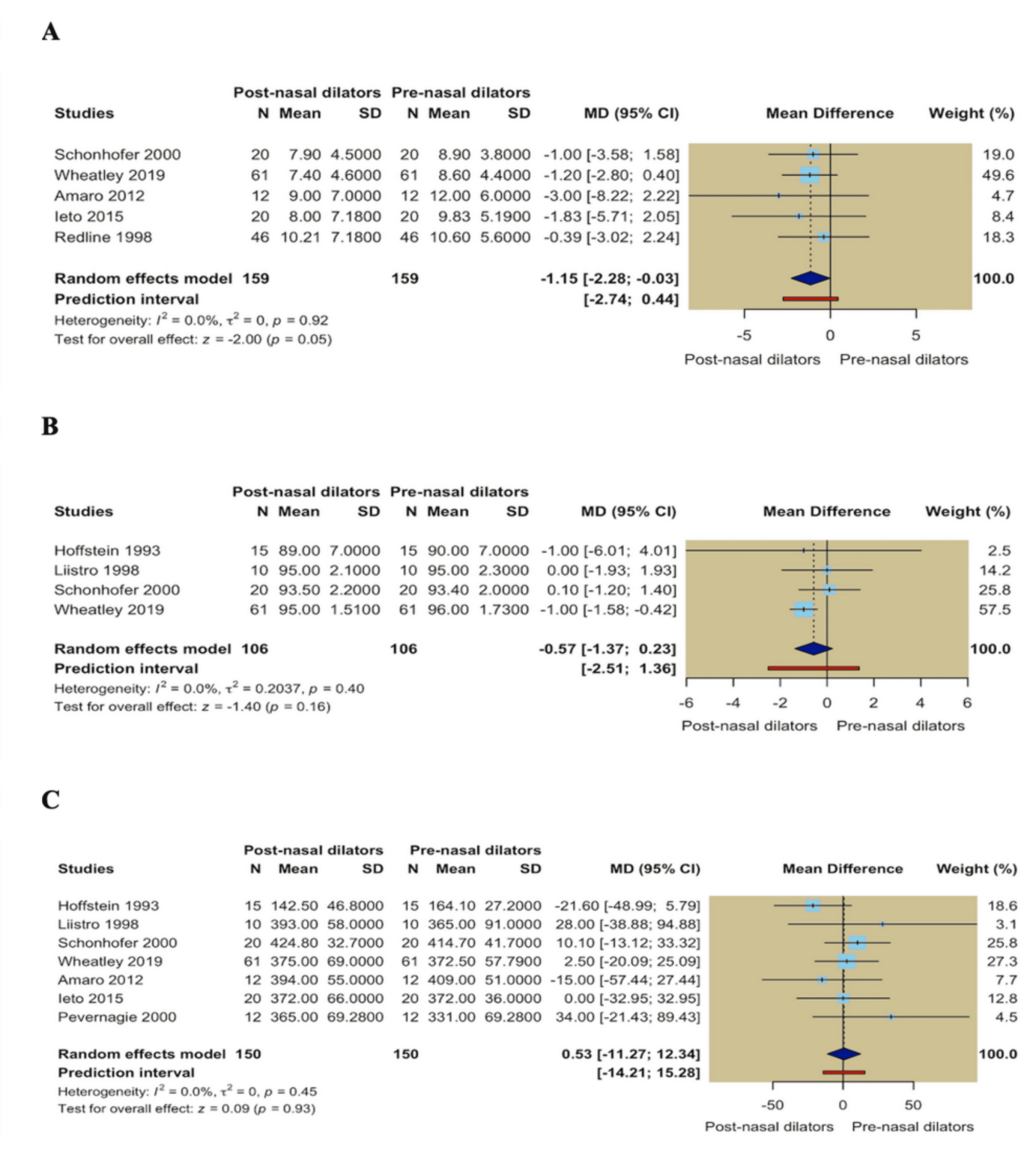
(A) Forest plot of Epworth Sleepiness Scale; (B) Forest plot of mean oxygen saturation; (C) Forest plot of total sleep time.

Single-arm meta-analysis: In single-arm analyses comparing post- versus pre-nasal dilator measurements, no significant within-group improvements were observed across sleep-disordered breathing severity outcomes. Pooled estimates showed no significant changes in AHI (MD = -0.03; 95% CI -1.92 to 1.87; *P* = 0.98), apnea index (MD = -0.64; 95% CI -3.97 to 2.69; *P* = 0.71), hypopnea index (MD = -1.91; 95% CI -5.53 to 1.71; *P* = 0.30), or snoring index (MD = -3.02; 95% CI -6.91 to 0.87; *P* = 0.13) (Figures 7A-7D). In the same context, daytime sleepiness assessed by the ESS showed no significant improvement following nasal dilator use (MD = -1.15; 95% CI -2.28 to -0.03; *P* = 0.05; *I*² = 0.0%). Similarly, mean oxygen saturation did not change significantly following nasal dilator use (MD = -0.57; 95% CI -1.37 to 0.23; *P* = 0.16; *I*² = 0.0%). TST remained comparable before and after intervention (MD = 0.53; 95% CI -11.27 to 12.34; *P* = 0.93; *I*² = 0.0%) (Figures 8A-8C).

Discussion

This systematic review and meta-analysis assessed the impact of nasal dilators on sleep-disordered breathing by examining changes in respiratory event frequency, sleep architecture, oxygenation, and patient-reported symptoms. Across both comparative and single-arm analyses, nasal dilators did not yield clinically meaningful improvements in apnea-hypopnea index, apnea index, hypopnea index, or snoring-related measures, nor did they significantly improve minimum nocturnal oxygen saturation or mean oxygen saturation. Similarly, measures of sleep continuity and architecture, including TST, sleep stage distribution, and rapid eye movement sleep parameters, remained largely unchanged following nasal dilator use.

The lack of improvement across objective sleep-disordered breathing outcomes in the present meta-analysis is consistent with prior evidence reporting no meaningful reductions in the apnea-hypopnea index or related polysomnographic parameters among patients with moderate to severe obstructive sleep apnea [26]. Similarly, prior meta-analysis highlighted that nasal dilators do not significantly affect AHI, lowest oxygen saturation, or snoring indices when evaluated across heterogeneous OSA populations, reinforcing the null effects observed in the current pooled analyses [14]. In a cohort of heavy snorers, external nasal dilation failed to produce consistent reductions in snoring duration or intensity and, in some cases, was associated with worsening of AHI in individuals without pronounced baseline nasal obstruction, further supporting the absence of objective benefit observed in this study [28].

Although subjective improvements in sleep quality and daytime sleepiness have been frequently reported, these findings were not accompanied by improvements in objective respiratory indices or sleep architecture in the present literature [26]. This dissociation between subjective and objective outcomes has been attributed to placebo effects and expectancy bias, particularly in non-blinded or run-in phases of clinical trials where perceived benefits were not substantiated by polysomnographic measurements [28]. Even when small changes in apnea index or daytime sleepiness were detected in prior studies, their magnitude was considered clinically negligible, especially when post-intervention values remained within ranges indicative of persistent disease burden [1,16]. Collectively, both the current results and the existing literature indicate that nasal dilators do not meaningfully modify the physiological severity or trajectory of sleep-disordered breathing, with any potential benefit likely limited to selected individuals with severe nocturnal nasal obstruction rather than the broader OSA population [28].

Nasal dilators function by widening the external nasal valve through lateral displacement of the nasal vestibular walls, which stabilize the valve and reduce inspiratory collapse at the nasal entrance [14]. This mechanical expansion decreases nasal airway resistance and increases inspiratory airflow by enlarging the cross-sectional area of the anterior nasal segment [8,38]. Furthermore, by lowering nasal resistance, dilators can normalize the balance between nasal and oral breathing during sleep, as mouth breathing is primarily driven by elevated nasal resistance [33].

However, these upstream mechanical effects are insufficient to prevent the downstream pharyngeal collapse that characterizes obstructive sleep apnea, explaining the lack of significant improvement in respiratory event indices [1]. Even substantial increases in nasal cross-sectional area translate into limited physiological benefit, as a 10% expansion yields only modest airflow gains that do not stabilize the collapsible pharyngeal airway [1]. In some cases, excessive nasal dilation may even be counterproductive by reducing expiratory braking and adversely affecting gas exchange [28].

Strengths and limitations

This systematic review and meta-analysis pose multiple key strengths. A comprehensive synthesis of both comparative and single-arm evidence was undertaken, which enaples the evaluation of nasal dilators across a broad range of objective polysomnographic outcomes, sleep architecture parameters, oxygenation metrics, and patient-reported symptoms. The inclusion of single-arm pre-post analyses complemented between-group comparisons and enabled assessment of within-subject changes, thereby maximizing the use of available evidence in a field characterized by small trials.

Nevertheless, several limitations warrant consideration. First, pooling populations with obstructive sleep apnea and primary snoring introduces clinical heterogeneity, as these conditions differ substantially in their underlying pathophysiology and disease severity. While justified by overlapping clinical presentation and shared use of nasal dilators, this approach may dilute subgroup-specific effects. Second, the inclusion of crossover and single-arm studies increases susceptibility to carryover effects, regression to the mean, and temporal confounding, particularly when washout periods were insufficient or poorly reported. Third, many included studies were small and underpowered, resulting in wide CIs and substantial heterogeneity across outcomes. Fourth, reliance on subjective outcomes in non-blinded designs increases the risk of expectancy and placebo effects, which may partially explain discordance between patient-reported improvements and objective polysomnographic findings. Finally, variability in nasal dilator type, duration of use, baseline nasal obstruction, and outcome definitions limited the ability to perform robust subgroup or dose-response analyses.

Collectively, these limitations highlight the need for cautious interpretation of pooled estimates and underscore the importance of adequately powered, well-controlled trials with standardized outcome reporting in future research.

Clinical implications and future directions

The findings of this systematic review and meta-analysis suggest that nasal dilators should not be considered an effective standalone therapy for the management of obstructive sleep apnea, as they do not produce meaningful improvements in objective measures of disease severity, sleep architecture, or oxygenation. In routine clinical practice, their use may be limited to selected patients, such as primary snorers or individuals with prominent nocturnal nasal obstruction, where symptomatic relief rather than physiological disease modification is the primary goal. Nasal dilators may also have a role as adjunctive tools in combination with established therapies, particularly continuous positive airway pressure, where they may improve nasal comfort or tolerance without directly influencing apnea severity.

Future research should focus on well-designed, adequately powered RCTs with clear separation of patient populations, including primary snorers, mild obstructive sleep apnea, and moderate-to-severe disease. Studies should incorporate standardized polysomnographic outcomes alongside validated patient-reported measures and ensure appropriate blinding and washout periods, especially in crossover designs. Further investigation is also warranted to identify phenotypic subgroups most likely to benefit from nasal dilation, such as patients with objectively documented nocturnal nasal obstruction, and to explore the adjunctive value of nasal dilators in multimodal treatment strategies.

## Conclusions

This systematic review and meta-analysis demonstrates that nasal dilators do not provide clinically meaningful improvements in objective sleep-disordered breathing severity, sleep architecture, or nocturnal oxygenation. Nasal dilators, therefore, appear to have a limited role in clinical practice, potentially serving as adjunctive or symptomatic interventions in selected individuals rather than as definitive therapy for OSA.
